# Hypoxia and growth factor profiling in high myopia: linking HIF-1α suppression and PDGF-BB activation to structural degeneration

**DOI:** 10.3389/fmed.2025.1705777

**Published:** 2025-12-08

**Authors:** Salvador Mérida, Enrique García-Gen, Carmen Desco, Amparo Navea, Francisco Bosch-Morell

**Affiliations:** 1 Department of Biomedical Sciences, Faculty of Health Sciences, Universidad Cardenal Herrera-CEU, CEU Universities, Valencia, Spain; 2 Instituto de la Retina y Enfermedades Oculares, Valencia, Spain; 3 Fundación de Oftalmología médica de la Comunidad Valenciana (FOM), Valencia, Spain

**Keywords:** high myopia, choroidal thickness, HIF-1α, PDGF-BB, VEGF, HGF, IL-6, hypoxia signaling

## Abstract

**Introduction:**

High myopia is associated with structural and molecular changes in the eye, particularly involving hypoxia-responsive and fibrotic mediators. Growing evidence points to a subset of growth factors as central mediators of these alterations. Among these, our findings identify HIF-1α and PDGF-BB as key contributors to the progression of myopia.

**Methods:**

This cross-sectional study included 88 Caucasian patients undergoing cataract surgery, categorized into control, low myopia, and high myopia groups based on axial length. Clinical assessments involved interferometric axial length measurement, swept-source OCT for choroidal thickness, and maculopathy classification following IMI guidelines. Aqueous humor samples were collected intraoperatively and analyzed using multiplex Quantibody arrays to quantify HIF-1α, VEGF, HGF, PDGF-BB, IL-6, and IL-17. Statistical analyses included Kruskal–Wallis tests with Bonferroni correction and correlation analyses to explore associations between molecular profiles and clinical severity.

**Results:**

Patients with high myopia exhibited significantly reduced choroidal thickness and increased axial length (*p* < 0.01), along with a higher prevalence of degenerative maculopathy (71.9%). Molecular profiling revealed lower levels of HIF-1α and VEGF (*p* < 0.01), and elevated levels of PDGF-BB (*p* < 0.05), HGF, and IL-6 (*p* < 0.01) in the high myopia group. HIF-1α levels showed inverse correlations with disease severity, while PDGF-BB correlated positively with fibrotic markers. No significant differences were observed in IL-17 levels.

**Discussion:**

For the first time, this study identifies significant alterations in HIF-1α and PDGF-BB levels in high myopia, with consistent correlations to key clinical parameters such as axial length, choroidal thickness, and maculopathy severity. These findings indicate an association suggesting a shift from adaptive hypoxia signaling toward fibrotic remodeling in high myopia. The suppression of HIF-1α and upregulation of PDGF-BB suggest a molecular imbalance that may be associated with structural degeneration. The integration of clinical imaging and aqueous humor profiling supports the potential of HIF-1α and PDGF-BB as biomarkers and therapeutic targets in pathological myopia.

## 1 Introduction

Myopia, commonly known as nearsightedness, affects approximately one-quarter of the global population and significantly impairs visual function. This condition is becoming increasingly widespread. The current estimated global prevalence of myopia is 30.5% (1). Projections suggest that by 2050, myopia will affect 40–50% of the world’s population (1, 2). In individuals with myopia, images are focused in front of the retina due to excessive curvature of the cornea or lens and elongation of the eye (3). Consequently, myopia is one of the most prevalent forms of refractive error. In certain types of myopia, excessive axial elongation of the eyeball results in thinning of the choroid, retinal pigment epithelium, and scleral layer (3, 4). Both genetic and environmental factors contribute to the development of myopia (5).

High myopia (HM) is typically defined as a refractive error ranging from ≥ − 5.0 D to −10.0 D (spherical equivalent), accompanied by axial elongation exceeding 26.0 mm (6, 7). HM is often referred to as pathologic myopia. This condition can lead to degenerative changes in the posterior segment of the eye, including posterior staphyloma, retinochoroidal atrophy, myopic maculopathy, or retinal detachment, which may result in blindness in some cases (6).

The pathogenesis of myopia involves a hypoxic environment (8) and dysregulation of multiple signaling pathways, including the involvement of various growth and inflammatory factors (9, 10). The role of HIF in cellular, tissue, and organismal adaptation to hypoxia remains an important and highly active field (11). Studies in mouse models (12) and human cell cultures (13) have shown that the scleral hypoxia-associated HIF-1α pathway plays a pivotal role in myopia development. Similarly, in a pilot study (*n* = 41), we demonstrated the concurrence of human myopia, oxidative stress, and hepatocyte growth factor (HGF), together with vascular endothelial growth factor (VEGF) (10). HGF is primarily associated with its neuroprotective role and may be involved in idiopathic epiretinal membrane growth (14), while VEGF is mainly linked to myopic choroidal neovascularization (15). In addition to VEGF, several other molecules participate in the development of retinal and choroidal neovascularization, including platelet-derived growth factor (PDGF) (16). However, research on PDGF in myopia development remains scarce. Inflammatory factors also play a significant role in the pathogenesis of myopia. Xiao et al. (17) demonstrated in a recent mouse model the activation of the NF-κB pathway and the upregulation of inflammatory cytokines such as TNF-α, IL-6, and IL-1β, indicating their involvement in scleral remodeling and myopia progression.

Inflammatory mediators, IL-6 and IL-17, were selected for analysis due to their established roles in neuroinflammation and tissue remodeling. IL-6 is a pleiotropic cytokine that modulates immune responses and has been implicated in neurodegenerative processes, including retinal degeneration (18). Elevated IL-6 levels have been associated with chronic inflammation and structural damage in various ocular and central nervous system disorders. IL-17, particularly IL-17A and IL-17F, is secreted by Th17 cells and innate immune cells, and is known to induce the expression of proinflammatory cytokines such as IL-6, IL-8, and CXCL1 through activation of NF-κB and MAPK pathways (19). These cytokines contribute to extracellular matrix remodeling and may play a role in the fibrotic changes observed in pathological myopia. The inclusion of IL-6 and IL-17 in this study aims to explore their potential involvement in the inflammatory microenvironment of the myopic eye and their interaction with hypoxia-related and fibrotic pathways.

In a healthy eye, the ciliary processes secrete aqueous humor (AH) into the posterior chamber. AH is a clear fluid present in both the anterior and posterior chambers of the eye. It flows into the anterior chamber and drains through the trabecular meshwork and Schlemm’s canal. AH provides nutrition, removes metabolic waste products, and supports the avascular ocular structures (20). Therefore, we aimed to study alterations in different growth factors (HIF-1α, PDGF-BB, VEGF, and HGF) and inflammatory factors (IL-6 and IL-17) found in AH, in conjunction with a clinical comparison between myopic and non-myopic patients.

## 2 Materials and methods

### 2.1 Experimental groups and patient selection

We conducted a cross-sectional study at the Instituto de la Retina y Enfermedades Oculares, Valencia, Spain, involving patients scheduled for cataract surgery. The study population consisted of patients undergoing cataract surgery, which allowed safe and ethical collection of aqueous humor samples during the procedure without additional invasive interventions. Cataract prevalence increases significantly with age, making this cohort representative and accessible for intraocular fluid sampling (21). Furthermore, previous studies investigating high myopia in cataract surgery patients have also focused on older populations, reflecting the natural epidemiology of cataract and the clinical overlap between high myopia and age-related lens opacities (22). This design ensured adequate sample sizes across myopia subgroups while minimizing ethical concerns. All participants provided written informed consent. The study received approval from the Ethics and Research Committee of CEU Cardenal Herrera University (CEI18/009) and was conducted in accordance with the Declaration of Helsinki.

AH samples were collected from 88 eyes of 88 patients undergoing eye surgery at the same institution. Inclusion criteria comprised patients with cataracts eligible for surgery, refractive myopia, and hyperopic or astigmatic defects below +0.75 D and −1.75 D, respectively. Exclusion criteria included concomitant ocular diseases that could interfere with the results (e.g., ongoing maculopathy other than myopic maculopathy, uncontrolled glaucoma requiring two topical medications, any stage of uveitis, venous or retinal arterial occlusions, diabetic retinopathy, hyperopia over +2 D, and astigmatism over −2 D). Patients with uncontrolled hypertension, diabetes, or hypercholesterolemia despite treatment were also excluded. Similarly, eyes with active choroidal neovascularization (CNV) were excluded following IMI classification criteria (23). However, some highly myopic eyes presented sequelae such as lacquer cracks or Fuchs’ spots, which were documented according to IMI standards. The potential influence of axial length on aqueous humor biomarker concentrations (‘dilution effect’) has been previously discussed (24), and this factor was considered during interpretation of the results. All participants were identified as Caucasian and presented with nuclear and/or cortical cataracts, as well as posterior subcapsular cataracts graded 2–3 according to the Lens Opacities Classification System III.

Experimental groups were categorized based on axial length (24–26). Eyes with an axial length exceeding 26 mm were classified as high myopia (HM). Those with axial lengths between 23.5 and 25.9 mm, presenting physiological myopia due to a mismatch between the refractive components of the eye, were classified as low myopia (LM). Eyes with axial lengths below 23.4 mm served as the control group (C). Since all patients had cataracts, refractive status was not used for classification to avoid bias from index myopia (27). Previous studies have shown that axial length is not associated with nuclear cataracts in myopic patients (28).

### 2.2 Clinical exploration and aqueous humor sample collection

All patients underwent a comprehensive ophthalmologic examination, which included ETDRS best-corrected visual acuity (BCVA), anterior segment evaluation using a slit-lamp, and binocular ophthalmoscopy. The presence or absence of staphyloma was assessed, and maculopathy was classified according to the International Myopia Institute (IMI) classification (23). Axial length was measured using interferometry (Zeiss IOLMaster 700^®^, Carl Zeiss Meditec AG, Jena, Germany), and subfoveal choroidal thickness was obtained using swept-source optical coherence tomography (SS-OCT) (TOPCON, Tokyo, Japan). Two observers (CD and AN) manually performed the measurements using a caliper provided by the software (version 9.30.003.02). They selected sections where the foveal depression was clearly visible and obtained one measurement per patient and observer (two measurements per eye in total) at the center of the fovea. The interobserver agreement was 95%.

AH samples were collected during cataract surgery following standard protocols. All patients received preoperative therapy, including antibiotic prophylaxis (Oftalmowell, UCB Pharma; an eyedrop solution containing gramicidin, neomycin, and polymyxin B) and local anesthesia (Colircusí, Alcon Healthcare; an eyedrop solution containing tetracaine and oxybuprocaine). The eyelids and eyelashes were sterilized, and 5% povidone-iodine was instilled into the conjunctival sac. Sterile adhesive dressing was applied to separate the lashes, the ocular surface was rinsed with saline solution, and paracentesis was performed at the planned surgical incision site using a sterile, single-use 30 G needle. This allowed the collection of a 120 μL AH sample into a 1 cc sterile disposable syringe. The surgery then proceeded according to standard protocol. The collected sample was transferred into an Eppendorf tube, rapidly frozen in liquid nitrogen, and stored at −80 °C until analysis.

### 2.3 Biochemical analysis

Growth factors (HIF-1α, PDGF-BB, HGF, and VEGF) and cytokines (IL-6 and IL-17) were quantified using commercially available Human Custom Quantibody^®^ Arrays. This platform is based on a multiplexed sandwich ELISA, which enables the simultaneous and precise determination of multiple cytokines and growth factors.

The following reagents and components were used for the Quantibody® arrays: Custom Array Glass Slide, Quantibody^®^ Sample Diluent, Wash Buffer I 20X (Lot #Q0362719), Wash Buffer II 20X (Lot #0411119), Custom Array Lyophilized Standard Mix, Custom Array Biotinylated Antibody Cocktail, Cy3-equivalent dye-conjugated Streptavidin, Slide Washer/Dryer, and Adhesive Film. All materials were sourced from RayBiotech, Peachtree Corners, USA.

The final signal was analyzed using an Axon GenePix scanner. Fluorescence data were extracted using the GAL file provided by the manufacturer (GenePix, ScanArray Express).

### 2.4 Statistical analysis

Data are presented as mean ± standard deviation (SD). Statistical analyses were performed using the commercially available software IBM SPSS version 29.0.2.0 (IBM Corp., Armonk, NY, USA).

Normality was assessed using the Kolmogorov–Smirnov test (*p* > 0.05). Intergroup differences were evaluated using a one-way analysis of variance (ANOVA) for normally distributed variables, with *post hoc* analysis as appropriate, or the Kruskal–Wallis test for non-normally distributed variables. Levene’s test was used to assess homogeneity of variances. When homogeneity was confirmed (*p* < 0.05), the Tukey test was applied as the post hoc test. When homogeneity was not confirmed, the T3 Dunnett test was used. For non-parametric variables, the Kruskal–Wallis test was adjusted using the Bonferroni correction.

An *a priori* power analysis was performed for a one-way fixed-effects ANOVA (*α* = 0.05; *β* = 0.20), using VEGF as the primary outcome. Pilot data from our previous study (10) indicated a large effect size (η^2^ ≈ 0.30; f ≈ 0.66), justifying a minimum of ~25 eyes per group. The final allocation (Control = 24; LM = 32; HM = 32) preserved ≥80% power for the omnibus comparison.

To evaluate the strength of association between two variables, Pearson’s or Spearman’s correlation tests were used, depending on data distribution. Statistical significance was defined as *p* < 0.05, with *p* < 0.01 considered highly significant. For each comparison, the corresponding test statistic (F for ANOVA or H for Kruskal–Wallis) was reported alongside the *p*-value to comply with best reporting practices.

## 3 Results

### 3.1 The clinical data recorded were consistent

The study included 88 patients, distributed as follows: 24 controls, 32 in the low myopia (LM) group, and 32 in the high myopia (HM) group. The average ages were 75.4 ± 6.3 years, 73.6 ± 10.1 years, and 69.8 ± 12.4 years, respectively. The overall sample consisted of 56% women and 44% men. No significant differences were found in age or sex distribution among the groups. Therefore, these variables were not considered confounding factors, and all patients were analyzed as a single cohort for both clinical and molecular comparisons.

Choroidal thickness, measured by optical coherence tomography, was significantly reduced in the HM group (113.91 ± 80.23 μm) compared to the control (237.01 ± 63.52 μm) and LM (210.22 ± 72.62 μm) groups, with a significance level of *p* < 0.01 (Table 1). Regarding axial length, both the LM (24.56 ± 0.79 mm) and HM (29.05 ± 3.32 mm) groups showed significant differences compared to the control group (22.65 ± 0.52 mm), with *p* < 0.01. Additionally, a significant difference was observed between the LM and HM groups (*p* < 0.01).

**Table 1:** Clinical characteristics of the 88 patients included in the study, all of whom were of Caucasian ethnicity.

Group	*N*	Age (years)	Axial length (mm)	Choroidal thickness (μm)
Control (C)	24	75.4 ± 6.3	22.65 ± 0.52	237.01 ± 63.52
Low myope (LM)	32	73.6 ± 10.1	24.56 ± 0.79**	210.22 ± 72.62
High myope (HM)	32	69.8 ± 12.4	29.05 ± 3.32^#^	113.91 ± 80.23^#^

Groups were classified as high myopia (HM), low myopia (LM), and control (C) based on axial length. Values are expressed as mean ± SD.**p < 0.01 vs. control group; #*p* < 0.01 vs. control and LM groups.

Retinal pathologies in the study cohort were classified according to the system established by the IMI, which defines five stages of myopic macular degeneration (see Table 2). In the control group, 21 eyes showed no signs of degeneration, while tessellated fundus patterns were observed in 3 cases. In contrast, the HM group exhibited a substantially higher prevalence of macular degeneration. Findings such as tessellated fundus, diffuse and patchy chorioretinal atrophy, macular atrophy, neovascularization, and lacquer cracks were identified in 23 eyes within this group (Table 2).

**Table 2:** IMI-based classification of maculopathies expressed as a percentage of the total study population (*N* = 88).

Degrees of maculopathy	C	LM	HM
No myopic degenerative retinal lesion	21	28	9
Tessellated fundus	3	3	4
Diffuse chorioretinal atrophy	0	0	2
Patchy chorioretinal atrophy	0	1	3
Macular atrophy	0	0	3
PLUS: NEOVASCULARIZATION/FUCHS’ SPOT	0	0	4
PLUS: LACQUER CRACKS	0	0	7

### 3.2 Clinical and biometric data show relevant correlations with growth factors and cytokines

The analysis revealed significant correlations among various clinical and biometric parameters, highlighting the complex interplay between molecular markers and ocular structural changes (Table 3). Notably, HIF-1α levels showed significant correlations with axial length (*ρ* = −0.479, *p* < 0.001), choroidal thickness (*ρ* = 0.324, *p* < 0.01), and maculopathy grade (*ρ* = −0.226, *p* < 0.05), suggesting a multifaceted role in myopic progression. Similarly, PDGF-BB levels demonstrated significant positive correlations with axial length (*ρ* = 0.243, *p* < 0.05) and maculopathy grade (*ρ* = 0.217, *p* < 0.05), indicating a potential contribution to structural retinal changes.

**Table 3:** Spearman’s correlation coefficients between clinical and biometric parameters.

Biomarker	Axial length (mm)	Choroidal thickness (μm)	Maculopathy grade
HIF-1α (pg/mL)	−0.499**	0.324**	−0.226*
PDGF-BB (pg/mL)	0.243*	−0.200	0.217*
VEGF (pg/mL)	−0.496**	0.179	−0.142
HGF (pg/mL)	0.344**	−0.261*	0.399**
IL-6 (pg/mL)	0.392**	−0.104	0.187
IL-17 (pg/mL)	0.079	0.031	−0.005

Statistical significance is indicated as follows: *p < 0.05, **p < 0.01.

In addition, VEGF levels were strongly correlated with axial length (*ρ* = −0.480, *p* < 0.001). HGF levels showed positive correlations with axial length (*ρ* = 0.344, *p* < 0.01) and maculopathy grade (*ρ* = 0.399, *p* < 0.01), and a negative correlation with choroidal thickness (*ρ* = −0.261, *p* < 0.05). IL-6 levels were positively correlated with axial length (*ρ* = 0.392, *p* < 0.01). In contrast, IL-17 levels did not show significant correlations with any of the measured parameters.

### 3.3 High myopic patients present altered growth factor levels in aqueous humor

**Figure 1:**
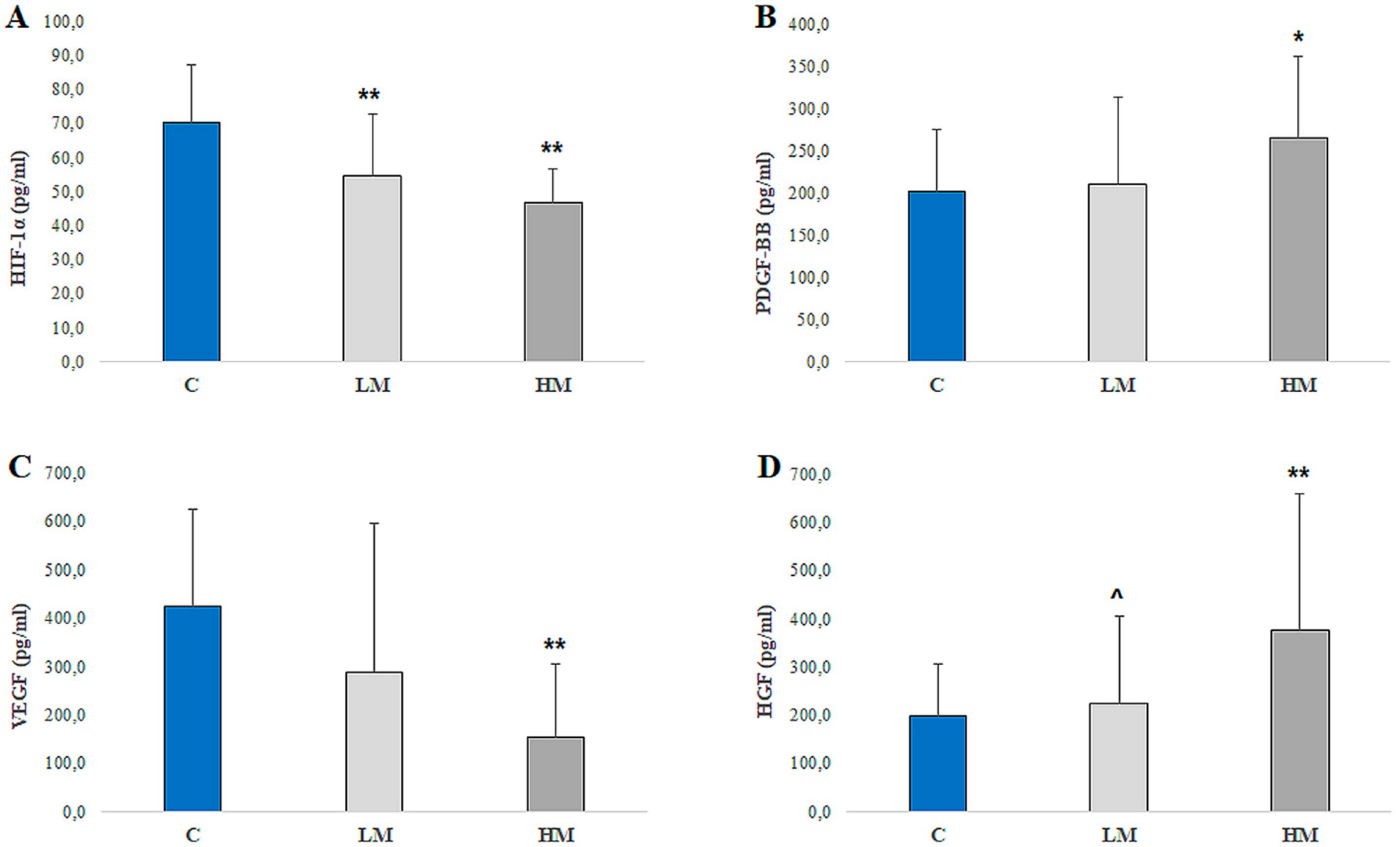
Concentrations of growth factors (**A**, HIF-1α; **B**, PDGF-BB; **C**, VEGF; and **D**, HGF) in aqueous humor (AH) samples collected from the Control group (*n* = 24), the Low Myopia group (*n* = 32), and the High Myopia group (*n* = 32). Values are presented as mean ± SE. **p < 0.01 compared to the Control group; *p < 0.05 compared to the Control group; ^*p* < 0.05 compared to the High Myopia group.

Patients in the HM group exhibited altered levels of the growth factors under investigation (Table 4 and Figure 1). Specifically, myopic patients demonstrated a dual trend: a reduction in AH HIF-1α levels (Figure 1A) and VEGF levels (Figure 1C), alongside an elevation in AH PDGF-BB levels (Figure 1B) and HGF levels (Figure 1D).

**Table 4:** Concentrations of growth factors and cytokines in aqueous humor (mean ± SD) across study groups.

Biomarker	Control	Low myopia	High myopia
HIF-1α (pg/mL)	70.29 ± 17.10	54.38 ± 18.21**	46.66 ± 10.01**
PDGF-BB (pg/mL)	202.50 ± 72.51	210,29 ± 102,91	264.59 ± 96.59*
VEGF (pg/mL)	422.79 ± 202.69	288,99 ± 306,36	154.03 ± 151.75**
HGF (pg/mL)	196.81 ± 110.33	223.31 ± 181.09	377.30 ± 281.83**
IL-6 (pg/mL)	38.84 ± 164.30	20.90 ± 37.08	82.14 ± 197.47**
IL-17 (pg/mL)	0.55 ± 1.97	0.24 ± 0.95	0.44 ± 2.15

Statistical significance is indicated as follows: *p < 0.05, **p < 0.01.

ANOVA revealed significant intergroup differences for HIF-1α (*F*(2,72) = 24.33, *p* < 0.001) and PDGF-BB (*F*(2,85) = 4.12, *p* = 0.019). *Post-hoc* Tukey tests confirmed that HIF-1α levels were significantly lower in LM and HM compared to controls (mean differences: Control vs. LM = 17.97, *p* = 0.001; Control vs. HM = 24.33, *p* < 0.001), while PDGF-BB was significantly higher in HM compared to controls (mean difference = 69.71, *p* = 0.014).

Notably, AH HIF-1α levels (Figure 1A) were significantly reduced in both the LM group (54.38 ± 18.21 pg./mL) and the HM group (46.66 ± 10.01 pg./mL) compared to the control group (*p* < 0.01). Similarly, PDGF-BB concentrations (Figure 1B) were significantly elevated in the HM group relative to controls (264.59 ± 96.59 pg./mL vs. 202.50 ± 72.51 pg./mL, *p* < 0.05), indicating a differential modulation of angiogenic signaling pathways.

In contrast, VEGF levels (Figure 1C) were markedly decreased in the HM group compared to the control group (154.03 ± 151.75 pg./mL vs. 422.79 ± 202.69 pg./mL, *p* < 0.01), suggesting a potential suppression of vascular permeability and neovascularization. Kruskal–Wallis tests confirmed significant differences for VEGF [H(2) = 14.99, *p* = 0.022] and HGF [H(2) = 21.55, *p* = 0.003], consistent with the trends shown in Figure 1. HGF concentrations (Figure 1D) were significantly higher in the HM group than in both the control group (377.30 ± 281.83 pg./mL vs. 196.81 ± 110.33 pg./mL, *p* < 0.01) and the LM group (377.30 ± 281.83 pg./mL vs. 223.31 ± 181.09 pg./mL, *p* < 0.05), highlighting a dose-dependent response in HGF expression. IL-6 also differed significantly across groups [H(2) = 20.46, *p* = 0.005], although variability was high, while IL-17 showed no significant differences [H(2) = 0.49, *p* > 0.05].

### 3.4 Studied growth factors show some interesting correlations

**Figure 2:**
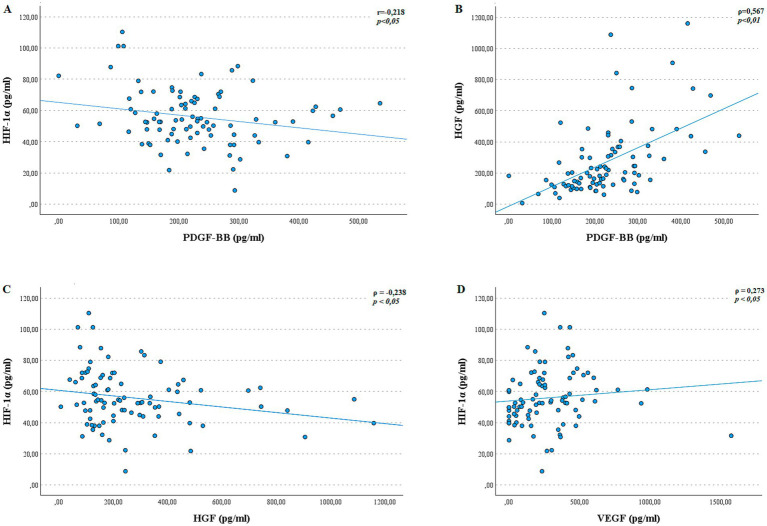
Correlations among the studied growth factors. Scatter plots showing Pearson’s or Spearman’s correlations between: **(A)** HIF-1α and PDGF-BB (*r* = −0.218, *p* < 0.05); **(B)** HGF and PDGF-BB (*ρ* = 0.567, *p* < 0.01); **(C)** HIF-1α and HGF (*ρ* = −0.238, *p* < 0.05); **(D)**, HIF-1α and VEGF (*ρ* = 0.273, *p* < 0.05).

HIF-1α, PDGF-BB, and HGF exhibited significant correlations among themselves. Specifically, HIF-1α levels were inversely correlated with PDGF-BB (Figure 2A, *R* = − 0.218, *p* < 0.05). Conversely, PDGF-BB showed a strong positive correlation with HGF (Figure 2B, *ρ* = 0.567, *p* < 0.01), while HIF-1α also demonstrated an inverse correlation with HGF (Figure 2C, *ρ* = −0.238, *p* < 0.05). Additionally, HIF-1α was positively correlated with VEGF (Figure 2D, *ρ* = 0.273, *p* < 0.05). These findings suggest a complex interplay among these growth factors, which may contribute to the underlying mechanisms of myopia.

No significant correlations were observed between the remaining growth factors. In particular, the associations between PDGF-BB and VEGF, as well as between HGF and VEGF, did not reach statistical significance (*p* > 0.05).

### 3.5 High myopic patients present altered IL-6 levels in aqueous humor

**Figure 3:**
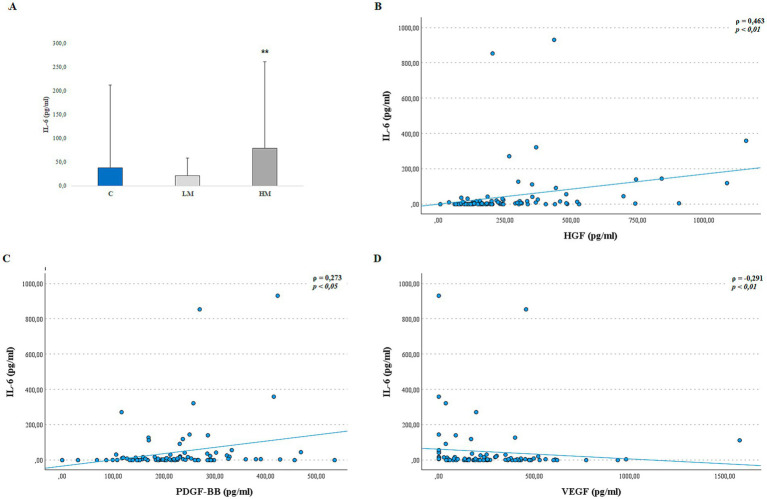
Key findings related to the IL-6 cytokine. **(A)** IL-6 concentrations in AH samples. Data are presented as mean ± SE. **p < 0.01 compared to the Control group. **(B–D)** Significant Spearman’s correlations between IL-6 and HGF **(B)**, PDGF-BB **(C)**, and VEGF **(D)**.

Patients included in the study exhibited considerable variability in cytokine levels, as reflected by the large standard deviations within groups. The Kruskal–Wallis test with Bonferroni correction revealed significantly elevated IL-6 levels in the high myopia (HM) group compared to the control group (Figure 3A: 82.14 ± 197.47 pg./mL vs. 38.84 ± 164.30 pg./mL, *p* < 0.01). No significant differences were observed in IL-17 levels among the groups (*p* > 0.05).

Interestingly, IL-6 showed significant correlations with several growth factors. Specifically, IL-6 was positively correlated with HGF (Figure 3B, *ρ* = 0.463, *p* < 0.01) and PDGF-BB (Figure 3C, *ρ* = 0.235, *p* < 0.05), while it was inversely correlated with VEGF (Figure 3D, *ρ* = −0.291, *p* < 0.01). These associations suggest a potential link between inflammatory and angiogenic pathways in the context of high myopia.

## 4 Discussion

### 4.1 Molecular correlates of myopic progression: linking growth factors and cytokines to ocular biometry

Our findings underscore the pivotal role of hypoxia- and growth-related signaling pathways in the pathophysiology of myopia. Among the biomarkers analyzed, HIF-1α and HGF exhibited the strongest correlations with clinical parameters (Table 3), indicating their potential association with axial elongation and myopic maculopathy, within a complex interplay of hypoxia, inflammation, and structural ocular changes.

The presence of HIF-1α in aqueous humor is intriguing, as this factor is primarily a nuclear transcription regulator. However, previous studies have reported detectable levels or stabilization of HIF-1α in ocular tissues and fluids under pathological conditions (29, 30). Possible sources include diffusion from ischemic retinal or choroidal tissues and secretion from anterior segment cells exposed to hypoxic stress. Recent reviews also highlight the role of HIFs in ocular ischemic diseases, supporting their relevance in the context of high myopia (56). These mechanisms may explain the measurable concentrations observed in our HM cohort.

The inverse association between HIF-1α and both axial length (*p* < 0.01) and maculopathy severity (*p* < 0.05), along with its positive correlation with choroidal thickness (*p* < 0.01), may be associated with a dysregulated hypoxic response in advanced myopia. While HIF-1α is typically upregulated under acute hypoxic conditions, chronic tissue remodeling in highly myopic eyes may be associated with impaired expression or downstream signaling (31). Recent transcriptomic and experimental studies identified scleral HIF-1α as a central regulator of hypoxia-driven myofibroblast transdifferentiation and extracellular matrix remodeling in myopic progression (32).

VEGF, a downstream effector of HIF-1α, also showed negative correlations with axial length (*p* < 0.01), which may indicate a compromised angiogenic response in highly myopic eyes, where VEGF expression is insufficient to counteract choroidal thinning and retinal ischemia. In contrast, HGF showed positive correlations with axial length and maculopathy severity (*p* < 0.01), and a negative correlation with choroidal thickness (*p* < 0.05). These findings support its association with processes such as scleral fibroblast proliferation and extracellular matrix turnover—processes implicated in axial elongation (33).

PDGF-BB also demonstrated significant positive correlations with axial length and maculopathy severity (*p* < 0.05), indicating its association with structural remodeling of the posterior segment. PDGF-BB is known to stimulate fibroblast proliferation and migration, and its elevated levels may be consistent with scleral thinning and biomechanical weakening. Supporting this, previous studies have shown that PDGF-BB promotes fibroblast proliferation and extracellular matrix production, processes central to scleral remodeling in myopia. For instance, Kang and Lee (34) demonstrated that PDGF-BB significantly enhances fibroblast activity and cytokine production in orbital tissues, highlighting its possible association with tissue remodeling and inflammation.

While myopic retinopathy is not traditionally classified as an inflammatory disease, a growing body of scientific evidence in recent years points to altered inflammatory biomarkers (35). In this context, interleukin-6 (IL-6), a key pro-inflammatory cytokine, has shown a significant positive correlation with axial length (*p* < 0.01), supporting the hypothesis that inflammation contributes to scleral remodeling and degenerative changes in myopia. This observation is consistent with recent findings implicating IL-6 and other inflammatory mediators in extracellular matrix disruption and immune signaling alterations associated with myopic progression (36).

### 4.2 Altered growth factor profiles and IL-6 in the aqueous humor of highly myopic eyes

Our results reveal a distinct molecular profile in the aqueous humor of high myopia (HM) patients, characterized by a significant reduction in HIF-1α (Figure 1A, *p* < 0.01) and VEGF (Figure 1C, *p* < 0.01), alongside elevated concentrations of PDGF-BB (Figure 1B, *p* < 0.05) and HGF (Figure 1D, *p*< 0.01). These findings indicate an association between hypoxia-related signaling and growth factor patterns in the myopic eye, rather than a direct mechanistic shift.

Although our samples were obtained from aqueous humor, several biomarkers identified are biologically linked to posterior segment pathology. VEGF and HGF may reflect hypoxia-driven signaling in the retina and choroid (37, 38), while nitrite accumulation indicates oxidative stress processes beyond the anterior chamber. Catalase activity provides indirect evidence of antioxidant imbalance relevant to posterior segment degeneration. Importantly, recent proteomic evidence has demonstrated that aqueous humor contains biomarkers directly associated with retinal diseases, including angiogenic and oxidative stress pathways (39).

The significant decrease in HIF-1α levels in both low myopia (LM) and HM groups (Figure 1A, *p* < 0.01) may be linked to advanced hypoxic conditions in the sclera, where prolonged tissue stress could influence HIF-1α stabilization or transcriptional activity (31). This interpretation aligns with previous studies by Zhao et al. (12, 32), which reported HIF-1α downregulation in advanced tissue remodeling stages. Notably, the hypoxia-induced HIF-1α/p53/miRNA-34a/Klotho axis in retinal pigment epithelial cells has been associated with subretinal fibrosis and choroidal neovascularization (40). More recently, Feng et al. (41) suggested that hypoxia, combined with hypoxia-activated platelets, may promote macrophage extracellular trap release and fibroblast transdifferentiation. These observations emphasize the broader inflammatory and cellular stress context in which growth factor alterations occur, indicating that the anterior segment molecular environment in high myopia reflects both impaired hypoxic signaling and enhanced fibrotic activity.

In this context, the elevated IL-6 levels observed in HM patients (Figure 3A, *p*< 0.05) may correspond to an active inflammatory microenvironment involving scleral macrophages. The high variability observed in IL-6 levels in our cohort is consistent with previous ocular studies, where IL-6 shows marked interindividual differences due to its pleiotropic nature and sensitivity to local microenvironmental factors (42–44). This variability has been attributed to the complex regulation of IL-6 expression by inflammatory and hypoxic stimuli, which may fluctuate depending on tissue remodeling and immune activation. Therefore, IL-6 should be interpreted as an exploratory marker rather than a definitive biomarker in the context of high myopia. IL-6 is a key cytokine involved in immune regulation and tissue remodeling. In a mouse model of form-deprivation myopia, IL-6 has been linked to macrophage polarization toward an M2 phenotype, which is associated with extracellular matrix remodeling and fibrotic progression (45). Liu et al. (46) reported that scleral hypoxia can induce IL-6 overexpression in human scleral fibroblasts, influencing their proliferation, differentiation, and apoptosis. Additionally, Droho et al. (42) observed that macrophage-derived IL-6 may stimulate choroidal angiogenesis through classical activation of IL-6R + macrophages. The correlations between IL-6 and HGF (Figure 3B, *p* < 0.01), PDGF-BB (Figure 3C, *p* < 0.05), and VEGF (Figure 3D, *p* < 0.01), as well as their associations with axial length (Table 3), support their involvement as correlates in fibrotic and degenerative changes.

Moreover, increased HGF levels in HM patients (Figure 1D, *p* < 0.01) are consistent with its reported role in promoting scleral fibroblast proliferation and matrix turnover—processes that have been linked to axial elongation in experimental models (33). Recent research also suggests that fibroblast growth factor 2 (FGF-2) may regulate scleral structure and influence myopia progression in guinea pig models (47). Evidence indicates that neuroinflammation and oxidative stress (55) are components of the molecular landscape in high myopia, with downregulation of neurotrophic growth factors and upregulation of oxidative markers contributing to tissue remodeling (26, 48).

The concomitant reduction in VEGF (Figure 1C, *p* < 0.01), a downstream effector of HIF-1α, supports the interpretation of an altered hypoxia–angiogenesis axis in high myopia. The reduced concentrations of HIF-1α and VEGF observed in highly myopic eyes appear consistent with previous reports indicating diminished angiogenic signaling in advanced myopia (49, 50). Several mechanisms may possibly contribute to this phenomenon: choroidal thinning and reduced vascular density could decrease oxygen and nutrient supply, while chronic hypoxic stress in degenerative tissues might impair HIF-1α stabilization and transcriptional activity (29). This scenario may lead to a relative downregulation of VEGF expression, reflecting a potential shift from an angiogenic to a fibrotic and atrophic microenvironment. These interpretations remain speculative and highlight the need for longitudinal studies to confirm whether these associations represent adaptive or pathological processes.

VEGF is essential for maintaining choroidal vasculature, and its suppression may be associated with choroidal thinning and ischemic stress observed in myopic degeneration (15). In contrast, the elevated PDGF-BB levels observed in the aqueous humor of highly myopic patients (Figure 1B, *p* < 0.05) may reflect fibrotic remodeling in the posterior segment. PDGF-BB has been shown to promote pericyte proliferation and survival via ERK signaling, and to modulate inflammatory responses through Akt pathways (51). Although these mechanisms have been primarily studied in the brain, they are relevant to the retina and may contribute to subretinal fibrosis in pathological myopia. Its upregulation in myopic eyes may indicate an association with scleral architecture changes in response to biomechanical stress. This interpretation is supported by our finding of an inverse correlation between PDGF-BB and HIF-1α (Figure 2A, *p* < 0.05), which may represent a compensatory pattern under hypoxic stress. Conversely, the strong positive correlation between PDGF-BB and HGF (Figure 2B, *p* < 0.01) suggests a possible interaction between these two growth factors in promoting fibroblast activation and extracellular matrix remodeling.

The significant intergroup differences identified in this study—particularly the suppression of HIF-1α and the upregulation of PDGF-BB in highly myopic patients—highlight a notable association with a shift in the molecular environment of the eye. These findings reinforce the potential translational relevance of aqueous humor analysis and suggest that future research should prioritize longitudinal studies to clarify temporal changes and their potential predictive value.

Interestingly, IL-17 levels were extremely low or undetectable across all study groups (Table 4), indicating that Th17-mediated immune responses are unlikely to be a major component of the aqueous humor microenvironment in high myopia. IL-17 is typically associated with autoimmune ocular diseases such as uveitis, where it promotes neutrophil recruitment and tissue damage (52–54). Its absence in our cohort suggests that Th17-mediated inflammation is unlikely to play a major role in the aqueous humor environment of high myopia. Instead, other pathways such as IL-6 signaling and growth factor-related remodeling may be more relevant in this context. This interpretation is tentative and aligns with previous reports showing negligible IL-17 expression in non-autoimmune degenerative conditions.

Finally, the interpretation of biomarker concentrations in highly myopic eyes should consider certain anatomical and pathological factors. The larger ocular volume associated with increased axial length may contribute to a ‘dilution effect,’ potentially reducing absolute cytokine levels. Although eyes with active CNV were excluded, a small proportion of patients exhibited sequelae such as lacquer cracks or Fuchs’ spots, which are unlikely to substantially influence VEGF concentrations but represent a source of heterogeneity. These aspects highlight the complexity of biomarker assessment in HM and should be addressed in future longitudinal studies.

## 5 Study limitations

While the present study offers valuable insights into the molecular landscape of high myopia, certain limitations should be considered. The cross-sectional design restricts the ability to evaluate temporal changes in biomarker expression. Furthermore, although the sample size was sufficient to detect significant differences, larger cohorts and longitudinal follow-up would be beneficial to validate these findings and assess their predictive value over time.

The potential influence of axial length on aqueous humor biomarker concentrations, including the so-called ‘dilution effect,’ should be acknowledged. Although all samples were collected under standardized conditions, the larger ocular volume in highly myopic eyes may partially affect absolute concentrations. Eyes with active choroidal neovascularization (CNV) were excluded, and only a small proportion of HM patients exhibited sequelae such as lacquer cracks or Fuchs’ spots, which are typical of advanced myopic degeneration. These cases were few and unlikely to substantially influence VEGF levels, but this potential confounding factor should be considered when interpreting the results.

## 6 Conclusion

High myopia is characterized by structural and molecular alterations, where HIF-1α and PDGF-BB show opposing patterns associated with disease severity. This study demonstrates statistically significant reductions in choroidal thickness and HIF-1α levels in HM patients, along with elevated concentrations of PDGF-BB, HGF, and IL-6 compared to emmetropic controls. The suppressed HIF-1α levels observed in severely affected eyes may indicate an impaired hypoxia response, while the upregulation of PDGF-BB and its positive correlations with fibrogenic cytokines suggest an association with tissue remodeling and inflammation. These findings support the relevance of integrating clinical imaging with aqueous humor profiling to improve diagnostic precision and to identify HIF-1α and PDGF-BB as potential biomarkers for pathological myopia. Further longitudinal studies are needed to clarify these associations over time.

## Data Availability

The datasets presented in this article are not readily available because the original contributions presented in the study are included in the article/supplementary material, further inquiries can be directed to the corresponding author. Requests to access the datasets should be directed to salvador.merida@uchceu.es.
